# Association of Novel Pathogenic Variant (p. Ile366Asn) in *PLA2G6* Gene with Infantile Neuroaxonal Dystrophy

**DOI:** 10.3390/ijms26010352

**Published:** 2025-01-03

**Authors:** Asma Naseer Cheema, Ruyu Shi, M. Ilyas Kamboh

**Affiliations:** 1 Children’s Hospital & The Institute of Child Health Multan, Multan 66000, Pakistan; 2 Department of Human Genetics, School of Public Health, University of Pittsburgh, Pittsburgh, PA 15261, USA; rus39@pitt.edu (R.S.); kamboh@pitt.edu (M.I.K.)

**Keywords:** variant, infantile neuroaxonal dystrophy, leukodystrophy, autosomal recessive, *PLA2G6*, iPLA2

## Abstract

A couple presented to the office with an apparently healthy infant for a thorough clinical assessment, as they had previously lost two male children to a neurodegenerative disorder. They also reported the death of a male cousin abroad with a comparable condition. We aimed to evaluate a novel coding pathogenic variant c.1097T>A, *PLA2G6*, within the affected family, previously identified in a deceased cousin, but its clinical significance remained undetermined. A 200 bp PCR product of target genome (including codon 366 of *PLA2G6*) was amplified followed by enzymatic digestion (*Mbo*I) and sequencing. Structural pathogenic variant analysis was performed using PyMOL 2.5.4. In RFLP analysis, the mutant-type allele produced a single band of 200 bp, and the wild-type allele manifested as two bands of 112 bp and 88 bp. The pathogenic variant was identified in nine family members, including two heterozygous couples with consanguineous marriages resulting in affected children. It was predicted to be deleterious by multiple bioinformatic tools. The substitution of nonpolar isoleucine with polar asparagine of iPLA2 (Ile366Asn) resulted in a eense pathogenic variant (ATC>AAC). A missense variant (p. Ile366Asn) in the *PLA2G6* gene is associated with clinically evident infantile neuroaxonal dystrophy, which is transmitted in an autosomal recessive pattern, and is also predicted to be dysfunctional by bioinformatic analyses.

## 1. Introduction

Infantile neuroaxonal dystrophy (INAD) constitutes a rare neurodegenerative disorder associated with *PLA2G6* gene mutation that exhibits a diverse spectrum of clinical presentation starting between six months and three years of age, characterized by progressive psychomotor regression, hypotonia, and gradually worsening stiffness in all limbs. Some children never achieve walking milestones, or they lose this ability shortly after gaining it. Common accompanying symptoms include the misalignment of the eyes (strabismus), involuntary eye movements (nystagmus), and damage to the optic nerve (optic atrophy). The disease progresses rapidly, leading to severe muscle stiffness, declining cognitive abilities, and worsening vision. Sadly, many affected children do not live past their first ten years [1,2,3].

This condition classically arises due to pathogenic alterations in the DNA sequence of phospholipase A2 group VI gene (*PLA2G6*), located on the chromosome 22q13.1 region, encoding a cytosolic Ca^2+^-independent phospholipase A2 protein (iPLA2), crucial for cell membrane stability [4,5]. Diagnosis relies on clinical presentation and neurophysiological, neuroradiological, and biopsy findings. Advancements in DNA sequencing have facilitated efficient genetic confirmation [6]. The diagnosis is confirmed through the identification of pathogenic variants within the *PLA2G6* gene via molecular genetic analysis. *PLA2G6* was identified as the causative gene in 2006, with over 150 reported cases, mostly classic INAD [7,8,9], and more than 200 pathogenic/likely pathogenic variants documented in the ClinVar Database (https://www.ncbi.nlm.nih.gov/clinvar/?term=%22PLA2G6%22%5BGENE%5D&redir=gene (accessed on 2 October 2024)).

Pakistan boasts a populace exceeding 240 million individuals (https://www.unfpa.org/data/world-population/PK (accessed on 1 October 2024)). The robust cultural architecture and varying socioeconomical fabric of Pakistani society prompt it to the have the highest prevalence of consanguineous unions globally, i.e., approximately 70% [10,11].

The cases of INAD are underreported in Pakistan because of the non-availability of genetic testing. In this study, we describe the inheritance of a novel pathogenic variant in *PLA2G6* associated with INAD in Pakistani kindred.

## 2. Results

### 2.1. Clinical Presentation

**Figure 1:**
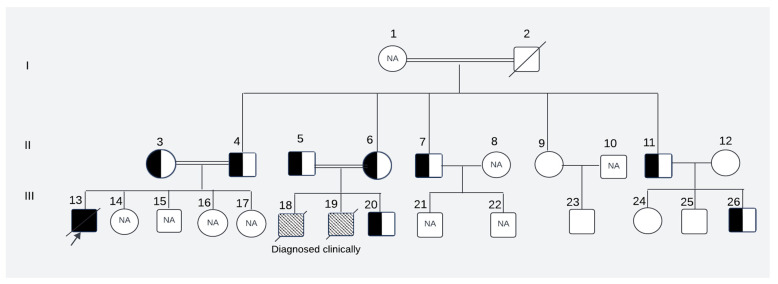
The pedigree of the affected family. NA: Samples were not available for these subjects; patterned box: not tested genetically but clinically diagnosed as cases of leukodystrophy; I, II, III: first, second and third generations of the family respectively; 1–2: Maternal grandparents of the presented child; 3–12: Maternal aunts, uncles with respective spouses of the presented child; 13–26: Maternal first cousins of the presented child(The description of symbols of pedigree is given in Supplementary Figure S1).

The perinatal histories of the subjects manifested unambiguous normalcy, characterized by unremarkable birth occurrences. All three affected children (III-13, III-18, III-19, Figure 1) were born at full term, with normal Apgar score and healthy birthweight.

They exhibited typical developmental trajectories until they reached the age of independent standing around 9–10 months of age. Notably, this milestone remained unattained and was accompanied by the subsequent regression of previously acquired developmental milestones. All three affected children (two siblings and their first cousin) displayed similar clinical presentations, including progressive muscle weakness, impaired motor skills that prevented independent standing and walking, hypotonia/hyperreflexia initially, hypotonia at a later stage, strabismus, nystagmus, and irritability. Tragically, all succumbed to death because of respiratory failure by the age of 9 years.

### 2.2. Genetic Analysis

**Figure 2:**
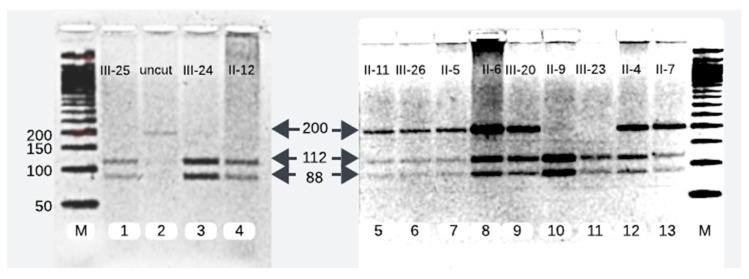
RFLP analysis of 200 bp PCR product. Lane 2 contains 200 bp uncut DNA. Lanes 1, 3, 4, 10, and 11 show the homozygous wild-type and lanes 5, 6, 7, 8, 9, 12, and 13 depict mutant heterozygotes of *PLA2G6* at c.1097T>A.

We successfully analyzed 12 samples of the affected family members. RFLP analysis of 7 of the 12 family members disclosed them as heterozygotes of the pathogenic variant (c.1097T>A). The pathogenic variant was characterized by a single band of 200 base pairs (bp), whereas the wild-type allele manifested as two separate bands of 112 bp and 88 bp. On gel electrophoresis, individuals carrying one mutant allele alongside one wild-type allele exhibited three distinct bands, whereas healthy subjects possessing two wild-type alleles displayed two bands (Figure 2).

**Figure 3:**
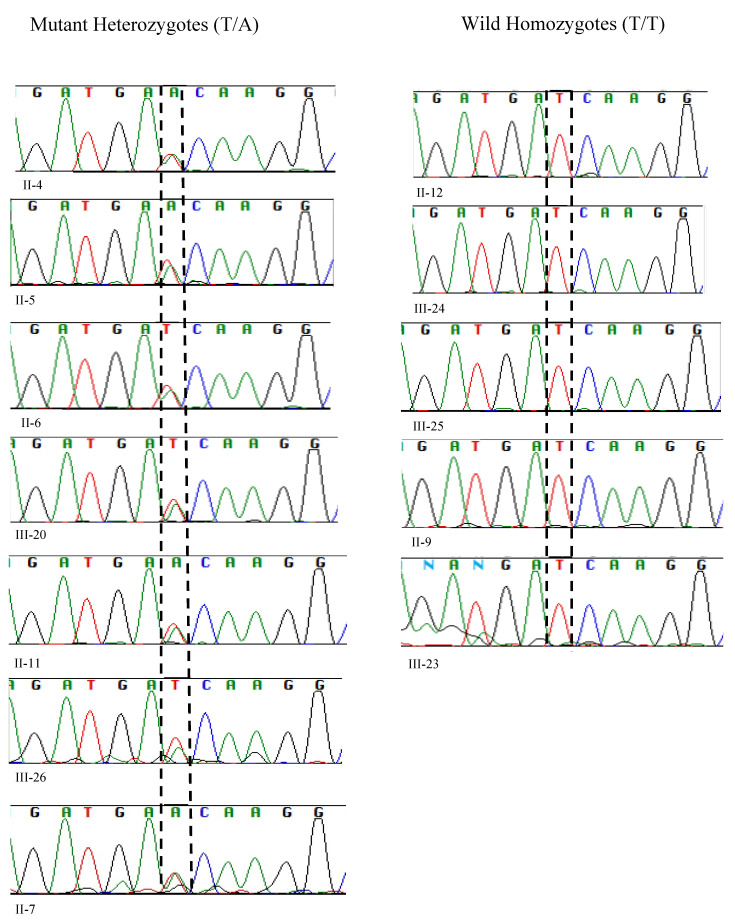
Sanger sequencing analysis of 12 samples unveiled 7 mutant heterozygotes, harboring the pathogenic allele T>A with two peaks at genomic position c.1097 of *PLA2G6*, (left-hand side), and 5 homozygotes of the wild allele with a single peak at c.1097 (T/T).

The sequencing results paralleled those obtained from RFLP analysis. Sanger sequencing revealed the presence of heterozygous alleles, with one allele being wild-type and the other mutant at position c.1097T>A (Gene Bank accession number NM_003560.4 with T>A of ATC) (Figure 3).

We reproduced the results of the proband’s father (II-4) and compared and matched his RFLP and Sanger sequencing results with other heterozygotes of the pathogenic variant (II-5, II-6, II-7, II-11, III-20, and III-26) (Figure 2 and Figure 3).

The Pakistani couple (II-5 and II-6) seeking the evaluation of INAD for their newborn child were heterozygous for the pathogenic variant and so was their third child (III-20) (Figure 2 and Figure 3).

Among the five maternal siblings in generation II, including the mother of the presented child, four were identified as heterozygotes of the pathogenic variant (II-4, II-6, II-7, and II-11; Figure 1, Figure 2 and Figure 3). Only one sister (II-9, Figure 1, Figure 2 and Figure 3) was homozygous for the wild-type allele. Despite carrying the mutant allele, all four siblings exhibited normal phenotypes and were free of any clinical manifestations of axonal dystrophy.

One maternal uncle and the mother (II-4 and II-6, Figure 1, Figure 2 and Figure 3) of the presented child, both of whom were heterozygous for the pathogenic variant, entered consanguineous marriages with their first cousins who were also heterozygous for the pathogenic variant (II-3, and II-5; Figure 1, Figure 2 and Figure 3). Notably, these two cousin marriages resulted in offsprings affected by INAD, indicating that the mutant allele had been inherited from both parents, which is consistent with the autosomal recessive mode of transmission of this pathogenic variant. One maternal uncle of the presented child who was heterozygous for the pathogenic variant (II-11) married a wild-type homozygous partner outside of the family, resulting in one offspring who is heterozygous for the pathogenic variant (III-26, Figure 1, Figure 2 and Figure 3). This case underscores the impact of consanguinity on the inheritance of autosomal recessive conditions such as axonal dystrophy.

### 2.3. Bioinformatic Analyses

**Figure 4:**
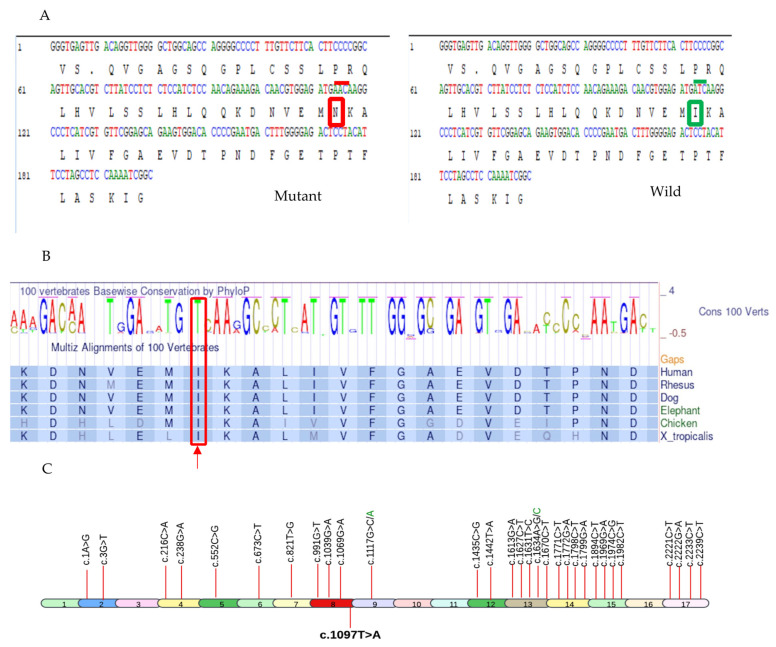
The illustration of pathogenic variant analysis of codon 366 using bioinformatics tools and sequence data from NCBI and UniProt databases. (**A**) The nucleotide substitution c.1097T>A resulted in the replacement of Ile (enclosed in green box on right side with overlying wild codon ATC under green line) with Asn (enclosed in red box on left side with overlying mutant codon AAC under red line) at codon 366 of *PLA2G6* (**B**) Thiamine(T) at c.1097 of *PLA2G6* gene is highly conserved across multiple species(enclosed in a red box and pointed to with an upward red arrow), underscoring its potential functional significance. (**C**) All known 32 pathogenic missense mutations associated with INAD, derived from ClinVar, are depicted in the *PLA2G6* gene at their respective exons. The mutation identified in this study (c.1097T>A) is highlighted in bold.

The genomic location was identified on Chr22: 38129543 corresponding to rsID: rs778225931. According to the gnomAD exome database, this variant is located on the exon 8 of *PLA2G6* gene and has a minor allele frequency (MAF) of 0.000004. Based on analyses from the NCBI and UniProt databases, a single nucleotide change from thymine to adenine at position c.1097 (c.1097T>A) has been identified, resulting in the substitution of isoleucine (Ile or I) with asparagine (Asn or N) at position 366 (p.Ile366Asn) (Figure 4A). This nucleotide is highly conserved across multiple species, indicating its potential significance (Figure 4B). 

This identified amino acid change was predicted as deleterious by five online bioinformatic tools (Table 1).

Substitution at position 366 from isoleucine (Ile or I) to asparagine (Asn or N) is predicted to affect the protein function with the SIFT score of 0.00, which is considered highly pathogenic. Median sequence conservation by SIFT algorithm was calculated as 3.00 for this amino acid position indicating that Ile is highly conserved at codon 366 across all species (Figure 4B) and its exchange with Asn is not predicted to be tolerable.

The other four tools also predicted it to be pathogenic: CADD (27.4, threshold > 15 for pathogenic variant); Polyphen-2 (0.92, threshold > 0.5 for pathogenic variant); REVEL (0.583, threshold ≥ 0.5 for pathogenic variant); and Alpha Missense (0.676, threshold > 0.5 for pathogenic variant).

Of >200 pathogenic/likely pathogenic mutations in *PLA2G6* gene submitted to ClinVar so far, we have demonstrated 32 pathogenic missense mutations in Figure 4C, (Supplementary Table S1). The novel 33rd pathogenic missense mutation (c.1097T>A) is also added in Figure 4C.

**Figure 5:**
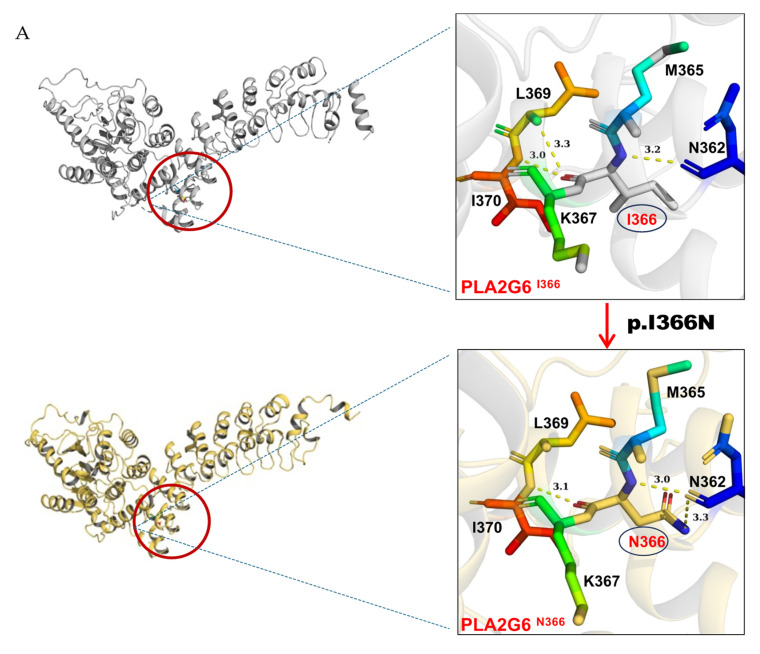

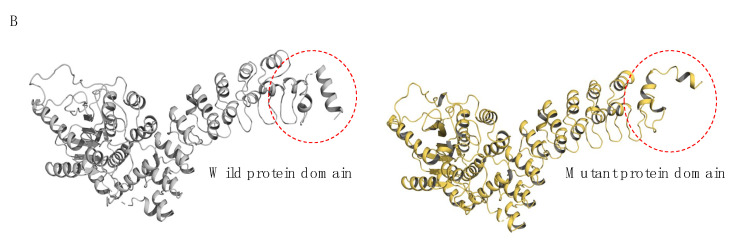
The graphical representation of structural pathogenic variant analysis of *PLA2G6* (iPLA2). (**A**) The structural impact of the I366N mutation was modeled based on the template of 6AUN derived from PDB by Swiss-model, and the mutant variant was modeled by PyMOL2.5. Structural prediction was performed with AlphaFold3. In the wild-type protein, residue I366, a hydrophobic (lipophilic) amino acid, formed three hydrogen bonds with neighboring residues N362, L369, and I370. In the mutated protein, I366 was replaced by N366, a polar (hydrophilic) amino acid, resulting in altered hydrogen bonding interactions: two bonds with N362 and one bond with I370. (**B**) The mutated structure exhibited poor overlap with the wild-type conformation, with pronounced distortions observed in the loop region comprising residues 81–114.

PyMOL structural analysis further supported the deleterious effects of the Ile366Asn pathogenic variant by demonstrating significant alterations in the protein’s 3D structure, which could disrupt its functional stability (Figure 5A).

The analysis revealed that the mutation at residue 366 of the protein caused significant changes in the three-dimensional structure. This region did not fully overlap with the wild-type protein, with notable structural distortions observed in the loop region of residues 81–114. These changes could potentially impair the protein’s function.

Moreover, the mutation significantly disrupted the protein’s hydrogen bond network. In the original structure, the nonpolar amino acid isoleucine (I366) interacted with neighboring residues L369, I370, and N362 via hydrogen bonds, contributing to the protein’s stability. However, upon the mutation of hydrophobic isoleucine (I366) to the polar asparagine (N366), the hydrogen bond between I366 and L369 was lost, while the hydrogen bond number between I366 and N362 increased from one to two (Figure 5A). These alterations in hydrogen bonding may lead to local structural instability, thereby affecting the overall function and stability of the protein, increasing the risk of misfolding or inactivation.

This substitution, involving a transition from a hydrophobic to a polar amino acid, disrupted the protein’s structural integrity. Hydrophobic residue I366 stabilized the protein core, whereas polar residue N366 was oriented toward the protein’s surface. This polarity shift significantly affected the protein’s secondary structure, particularly the α-helix a and its overall tertiary conformation.

Structural prediction analysis by Alpha fold3 revealed that the mutation at residue 366 induced substantial alterations in the three-dimensional architecture. The mutated structure exhibited poor overlap with the wild-type conformation, with pronounced distortions observed in the loop region comprising residues 81–114 (Figure 5B). These changes underscore the critical role of I366 in maintaining the native structural framework of the protein.

## 3. Discussion

We have characterized Pakistani kindred with a rare pathogenic variant in the *PLA2G6* gene segregating with INAD with an autosomal recessive mode of inheritance. This pattern is characterized by a 25% risk of having an affected child in each pregnancy when both parents are heterozygous for the pathogenic variant and highlights the critical importance of genetic counseling for families with a history of autosomal recessive disorders in this part of the world where consanguineous marriages are common.

INAD itself is a single-gene disorder, but it is a part of a broader group of diseases called *PLA2G6*-associated neurodegeneration (PLAN) [12]. INAD typically manifests clinically between 6 months and 3 years of age. In our case, the affected children were clinically diagnosed around the age of 1 year, when they exhibited an inability to stand or walk independently. The uniform clinical presentation of INAD in the affected children within this family underscores the disorder’s characteristic phenotype, which aligns with the clinical features described in other studies [1,2,3,7,8,9,13]. This consistency in symptomatology supports the notion of a recognizable and predictable clinical course for INAD, which can aid in early diagnosis. INAD is caused by different mutations within the *PLA2G6* gene. Over 200 pathogenic/likely pathogenic variants (missense, nonsense, frameshift, and splice site mutations) have been identified in this single gene. We have displayed all known pathogenic missense mutations associated with INAD in Figure 4C. Each of these mutations affect the *PLA2G6* function yet all lead to the clinical features of INAD.

The affected family’s situation further illustrates the challenges faced in regions with limited access to genetic testing facilities. The tragic loss of three male children to INAD emphasizes the dire consequences of not having access to early genetic diagnosis and intervention. The fact that two children, despite being phenotypically healthy, were identified as heterozygous for the pathogenic variant, highlights the potential for genetic screening to provide crucial information that can inform family planning and prevent future occurrences of the disorder by avoiding first-cousin marriages. Infantile neuroaxonal dystrophy is a very rare neurodegenerative disorder with no specific incidence data available for Pakistan. The absence of a patient registry for genetic diseases in Pakistan, including rare disorders like INAD, poses significant challenges for healthcare providers, researchers, and policymakers. The lack of comprehensive epidemiological data underscores the necessity for detailed research and data collection to understand the prevalence of such rare disorders in different regions in the country.

In our study, the single-nucleotide substitution at c.1097T>A produced a nonfunctional A2 phospholipase, an enzyme type that facilitates the liberation of fatty acids from phospholipids [14,15,16]. The sequence of this enzyme is divided into three domains: an amino terminal, Ankyrin, and catalytic domain [17]. Ankyrins are present in a multitude of proteins and have evolved into an extremely precise structural framework for recognizing proteins [18]. In various proteins, clusters of Ankyrins can align adjacent to each other, creating elongated linear formations. Within these formations, a hydrophobic core, comprising five conserved amino acids, maintains the cohesion of the helical repeats [19]. While the specific amino acids may vary, the overall three-dimensional structure of the Ankyrin remains remarkably consistent [20]. The altered hydrophobicity resulting from the Ile366Asn substitution may lead to misfolding or aggregation of the protein, impairing its function and contributing to cellular dysfunction. Isoleucine is highly conserved across species, and the substitution of this amino acid with asparagine at codon 366 (Ile366Asn) in the *PLA2G6* gene can lead to detrimental effects. Dysregulated phospholipid metabolism can result in increased oxidative stress within neurons [21,22,23]. Neuronal cells are highly susceptible to oxidative damage due to their high content of unsaturated fatty acids. The accumulation of oxidized phospholipids can lead to lipid peroxidation, the generation of reactive oxygen species, and subsequent damage to cellular membranes, proteins, and DNA, ultimately contributing to neuronal dysfunction and death. Phospholipids are also critical for maintaining mitochondrial structure and function [24]. The dysregulation of phospholipid metabolism may impair mitochondrial membrane integrity, disrupt electron transport chain activity, and compromise ATP production, leading to mitochondrial dysfunction and bioenergetic failure in neurons. Phospholipids also serve as precursors for signaling molecules involved in intracellular signaling pathways. Dysregulated phospholipid metabolism can disrupt the production of these signaling molecules, leading to aberrant cellular signaling and impaired neuronal function. This dysregulation may contribute to synaptic dysfunction, neurotransmitter imbalance, and altered neuronal excitability, ultimately leading to neurodegeneration [25]. Cumulative damage resulting from disrupted phospholipid metabolism, oxidative stress, mitochondrial dysfunction, and impaired neuronal signaling can ultimately lead to neuronal death. Neuronal loss contributes to the progressive neurodegeneration observed in affected individuals, resulting in the characteristic clinical features of the disorder, including movement abnormalities, cognitive decline, and neurologic dysfunction.

The identification of a novel mutation within this genetic region underscores the critical role of the *PLA2G6* gene in natural neurological development. In this study, we identified a pathogenic variant in the *PLA2G6* gene within a consanguineous family. This discovery holds significant implications for genetic counseling, screening, and preventive initiatives in consanguineous populations. By explaining the inheritance pattern and the associated risks, we guided the family in understanding the likelihood of passing on the genetic condition to future generations. Our data support the need for preventive measures, such as promoting genetic testing before marriage, raising awareness about the risks of consanguinity, and providing access to genetic counseling services. It can also inform public health policies focused on reducing the incidence of inherited diseases within consanguineous populations by encouraging proactive genetic testing and offering early interventions. Systematic genetic screening for known pathogenic variants facilitates early diagnosis and informed family planning decisions, potentially reducing the occurrence of genetic diseases in future generations.

The study broadened our comprehension of the genetic underpinnings of INAD. Furthermore, ongoing research into the molecular mechanisms governing INAD pathogenesis holds promise for identifying potential therapeutic targets conducive to intervention and treatment advancement.

## 4. Materials and Methods

### 4.1. Case Description

A Pakistani couple seeked medical advice at the Children’s Hospital, Multan, Pakistan, for their newborn offspring (presented child: III-20; Figure 1).

The couple (II-5 and II-6; Figure 1) had a cousin marriage and previously experienced the loss of two male children (III-18 and III-19; Figure 1), both diagnosed of having axonal dystrophy through clinical and neuroimaging evaluations. The family history revealed that a cousin (III-13, Figure 1) of the presented child from his maternal line and living abroad also succumbed to a similar ailment. The cousin’s parents (II-3 and II-4; Figure 1) were also related by consanguinity. The medical records of this cousin, who, along with his parents, underwent genetic testing abroad, were obtained. The records revealed that the cousin who later passed away was homozygous for the pathogenic variant at exon 8 position c.1097T>A of the *PLA2G6* gene, and both of his parents were heterozygous for the pathogenic variant. Since this variant had not been reported previously, it was considered of undetermined clinical significance and was not investigated further. To ascertain whether the newborn of the Pakistani couple had this condition and to assess whether this pathogenic variant was indeed the cause of INAD in the family, we reached out to all maternal uncles, aunts, their spouses, and children of the presented child. The study was approved by the University of Pittsburgh Institutional Review Board.

### 4.2. Clinical Data and Specimen Collection

The distribution of subjects by disease status, sample availability for genetic testing, and availability of genetic records is shown in Supplementary Figure S2)

Of the 22 living members of the family shown in Figure 1, we were able to collect saliva samples using 4 OGR-500 DNA saliva kits from 12 family members after obtaining written informed consent. Subject I-1 was too old and weak to give the sample. We could not obtain consent for genetic testing from 8 subjects (II-8, II-10, III-14, III-15, III-16, III-17, III-21, and III-22). The parents of the affected proband provided us with the genetic testing record of the affected proband (III-13) as well as their own genetic testing records (II-3 and II-4), while only clinical records were available for affected deceased children who were residents of Pakistan (III-18 and III-19). Rest of the family members were inquired and clinically assessed by the physician.

### 4.3. Laboratory Testing

DNA extraction from saliva was performed using the prepIT.L2P DNAgenoTeK kit. The samples were incubated at 50 °C for two hours. The entire saliva sample of each family member was transferred to a 15 mL conical tube. The sample volumes were noted, and 1/25th volume of prepIT.L2P was added to each sample and mixed by vortexing for few seconds. The samples were incubated for 10 min followed by centrifugation at room temperature for 10 min at 3500× *g*. The supernatants were carefully transferred to a fresh 15 mL tube. The pallets were discarded. A 1.2× volume of 99% ethanol was added to the clear supernatant of each sample, and all sample tubes were inverted gently for 10 times. The samples were allowed to stand at room temperature for 10 min to allow the DNA to fully precipitate. The samples tubes were centrifuged at 3500× *g* for 10 min. This time the supernatants of all samples were removed carefully with the help of pipette and discarded without disturbing the DNA pellet. A 1 mL volume of 70% ethanol was added to each tube without disturbing the pellet and allowed to stand for 1 min at room temperature. After swirling it gently, the ethanol was removed. The pellets in each tube were allowed to stand at room temperature until they dried. A 500 μL volume of TE solution was added to each sample, and it was allowed to stand at room temperature for two days. The samples were gently vortexed, transferred to a 1.5 mL tube, and centrifuged at room temperature for 15 min at 15,000× *g*. The supernatant of each sample was transferred to a fresh 1.5 mL tube and stored at −20 °C for long-term storage and subsequent application.

Primers (5′-GCCGATTTTGGAGGCTAGG-3′; 5′-GGGTGAGTTGACAGGTTGG-3′) were designed to amplify the genomic region spanning from position 38,129,458 to 38,129,657 on the sense strand within exon 8 of the NM_003560.4 transcript, utilizing the genomic sequence of NC_000022.11 (Chromosome 22 Reference GRCh38.p14 Primary Assembly). The resultant 200 bp PCR product was confirmed through agarose gel for all samples. To discern the homozygous and heterozygous states at this locus, restriction fragment length polymorphism (RFLP) analysis was employed using the smart cut enzyme *Mbo*I (5′… GATC …3′; 3′…CTAG …5′). The sample of proband’s father (II-4) was used as a positive control as it had previously been sequenced for this pathogenic variant and identified as heterozygous for this pathogenic variant.

The accuracy of the enzyme digestion outcomes was corroborated through Sanger sequencing, and the sequencing results were analyzed using the Sequencher software (v.5.4.6).

### 4.4. Bioinformatic Tools

#### 4.4.1. Genomic Position and Protein Domain

UniProt (https://www.uniprot.org (accessed on 12 September 2024)) [26], ClinVar (http://www.ncbi.nlm.nih.gov/clinvar/ (accessed on 2 October 2024)) [27], and gnomAD v4.0 (https://gnomad.broadinstitute.org/ (accessed on 12 September 2024)) [28] databases were utilized to identify the genomic position and specific protein domain affected by the pathogenic variant.

#### 4.4.2. Pathogenic Variant Annotation

The missense variant was assessed for its annotation using multiple in silico tools, including SIFT (http://sift.jcvi.org/ (accessed on 6 September 2024)) [29], Polyphen-2 (http://genetics.bwh.harvard.edu/pph2/ (accessed on 13 September 2024)) [30], CADD (http://cadd.gs.washington.edu (accessed on 23 September 2024)) [31], REVEL (https://sites.google.com/site/revelgenomics/ (accessed on 23 September 2024)) [32], and Alpha Missense (https://alphamissense.hegelab.org/ (accessed on 23 September 2024)) [33]. The functional explanation of these tools is described in Supplementary Table S2.

#### 4.4.3. Structural Modeling of Pathogenic Variant and Prediction

The structural modeling of the pathogenic variant was performed after deriving its basic structure from Protein Data Bank (https://www.rcsb.org/ (accessed on 2 October 2024)) [34]. The *PLA2G6* chain A structure was modeled on the crystal structure of *PLA2G6* (6AUN). The three-dimensional mutated structures on I366 residue were predicted by Swiss-model [35], and the potential impact of the pathogenic variant was analyzed by PyMOL 2.5.4 (PyMOL Software, Inc. San Diego, CA, USA). Hydrophobic interaction analysis was conducted with the measurement plugin. After obtaining the draft, Adobe Illustrator 2020CC was used for combining and annotating the images. Structural prediction was performed with AlphaFold3, selecting the best model based on pLDDT values as the experimental template [36].

## Figures and Tables

**Table 1:** The evaluation of the missense mutation (Ile366Asn) for its potential biological impact on protein function using various in silico prediction tools.

In Silico Tools	Threshold	Score	Predicted Effect
SIFT	≤0.05	0.00	Pathogenic
CADD	>15	27.4	Pathogenic
PolyPhen-2	>0.5	0.92	Pathogenic
REVEL	≥0.5	0.58	Pathogenic
Alpha missense	>0.5	0.68	Pathogenic

## Data Availability

The data gathered and examined for this manuscript are provided in the manuscript. The data are also submitted to the ClinVar submission portal—NCBI (https://www.ncbi.nlm.nih.gov/clinvar/submitters/509752 (accessed on 28 October 2024)). The summary report of successfully processed record can be accessed using the following link: https://submit.ncbi.nlm.nih.gov/api/2.0/files/axnklzyc/sub14800741__100__submitter_report_b.txt/?format=attachment (accessed on 28 October 2024).

## Supplementary Materials

The following supporting information can be downloaded at: https://www.mdpi.com/article/10.3390/ijms26010352/s1.
