# Supplementary material for: Association of Novel Pathogenic Variant (p. Ile366Asn) in PLA2G6 Gene with Infantile Neuroaxonal Dystrophy
Source: Int J Mol Sci. 2025 Jan 3;26(1):352. doi: 10.3390/ijms26010352 (PMC11721680; doi:10.3390/ijms26010352)
Supplement: Supplementary file 1 [file ijms-26-00352-s001.zip › ijms-3401320-supplementary.pdf]

Supplementary Table S1: Bioinformatic tools and their functional explanation

**Bioinformatic tools**	**Functional Explanation**
SIFT	It provides the position of the amino acid in the reference protein, denoted by Seq Rep, which represents the fraction of sequences containing one of the basic amino acids. A higher fraction (closer to 1.00) indicates the position is highly aligned and we expect dependable prediction at that position. The SIFT median, which spans from 0 to 4.32, ideally falls within the range of 2.75 to 3.5, reflecting the conservation and diversity of sequences utilized for prediction.
CADD	The predicted effect on the amino acid sequence is assessed using the CADD algorithm, with a "CADD-Phred" score ranging from 0 to approximately 99. A variant is typically considered deleterious if its score exceeds 15, which a higher score indicating a more likely damaging effect on the protein function. The score is calculated from conservation metrics and functional predictions.
PolyPhen-2	PolyPhen-2 evaluates amino acid substitutions by estimating their possible impact on the structure and function of a protein from evolutionary conservation, protein structure, and functional domains. It assigns a score between 0 and 1, with a higher score indicating likely damaging. Scores between 0.15 and 0.85 are categorized as "possibly damaging", and scores above 0.85 are "probably damaging".
REVEL	REVEL combines scores from various individual prediction algorithms to estimate the deleteriousness of missense variants. It assigns a score between 0 and 1, with higher scores indicating a higher likelihood that the variant is pathogenic. A typical threshold for considering a variant deleterious is ≥ 0.5, though the threshold may vary depending on the clinical or research content. It integrates data from tools such as SIFT, PolyPhen-2, and MutationTaster, among others, and prioritizes variants that are likely disrupt protein function based on multiple lines of evidence.
AlphaMissense	AlphaMissense predicts the effect of amino acid substitutions using a deep learning approach, trained on evolutionary and structural data. The score

	ranges from 0 and 1, where scores closer to 1 indicate a higher likelihood that the substitution will have a deleterious effect on protein function.

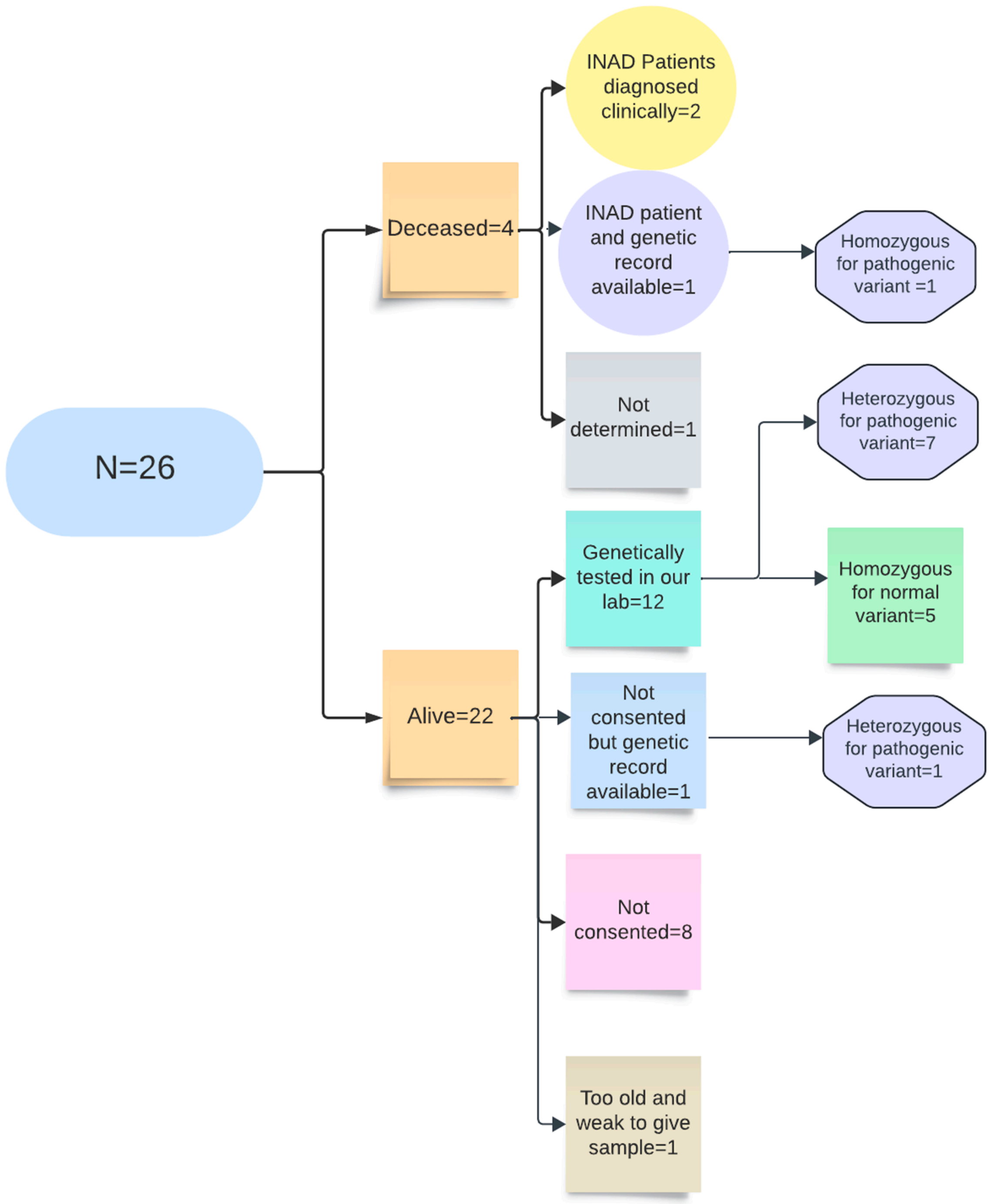

Supplementary Figure S1: Distribution of subjects by disease status, sample availability, and genetic testing

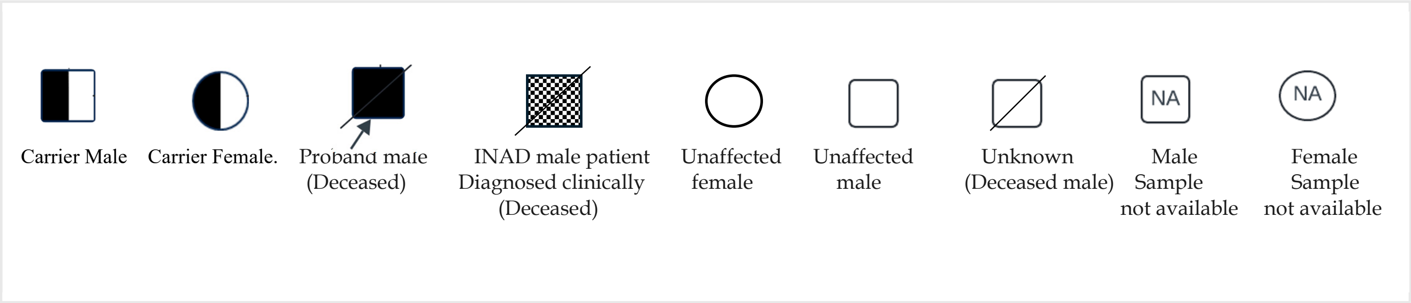

Supplementary Figure S2: Description of symbols used in pedigree
